# Modification of Chabazite Using Hexadecyltrime-Thylammonium Bromide (HDTMA-Br) for Chromium(VI) Removal from Water Solutions

**DOI:** 10.3390/ma18122897

**Published:** 2025-06-18

**Authors:** Agata L. Skwarczynska-Wojsa, Paulina Sobolewska, Marcin Chutkowski, Jolanta Warchol

**Affiliations:** 1 Department of Water Protection and Purification, Faculty of Civil Environmental Engineering and Architecture, Rzeszow University of Technology, 35-959 Rzeszow, Poland; psobolew@prz.edu.pl; 2 Department of Chemical and Process Engineering, Faculty of Chemistry, Rzeszow University of Technology, 35-959 Rzeszow, Poland; marcin.chutkowski@prz.edu.pl; 3 Department of Advanced Material Technology, Faculty of Chemistry, Wroclaw University of Science and Technology, 50-372 Wroclaw, Poland; jolanta.warchol@pwr.edu.pl

**Keywords:** chabazite-zeolite, sorption, chromium Cr(VI), kinetics, HDTMA-Br, environmental pollution

## Abstract

Chabazite, a tectosilicate mineral, belongs to the zeolite group and has been widely used for the adsorptive removal of a number of cationic contaminants from the aqueous phase. However, a negatively charged chabazite surface can be altered by chemical modification in order to change its adsorption abilities towards anions. This study reports the potential for the removal of hexavalent chromium ions from aqueous solutions by modified chabazite. In this regard, natural chabazite was modified by the immobilization of HDTMA-Br to achieve double-layer coverage on its surface, defined as the double external cation exchange capacity. Next, a batch adsorption system was applied to study the adsorption of inorganic Cr(VI) anions from aqueous solutions. The process equilibrium was described by 11 theoretical isotherm equations, while 6 adsorption kinetics were represented by four models. Among those tested, the most appropriate model for the description of the studied process kinetics was the pseudo-second order irreversible model. The obtained results suggest that Cr(VI) adsorption takes place according to a complex mechanism comprising both Langmuir-type sorption with the maximum adsorption capacity of modified chabazite, approx. 9.3–9.9 mg g^−1^, and the trapping of Cr(VI) inside the capillaries of the amorphous sorbent, making it a viable option for water treatment applications.

## 1. Introduction

Today, intensive industrial development brings many economic benefits, however, on the other hand, it contributes to irreversible negative environmental changes due to the discharge and accumulation of toxic substances such as heavy metals, including chromium compounds. In principle, the toxicity of chromium is determined mostly by the oxidation states ranging from 2 to +6, however the trivalent(III) and hexavalent(VI) states of chromium predominate in the environment and pose the risk of very serious negative health effects. In essence, hexavalent chromium Cr(VI) poses a hazardous risk to public health and all living organisms, and its potential to cause cancer has also been proven. Therefore, the World Health Organization classified Cr(VI) as a Group I carcinogen [1]. According to the European Union’s directive 2020/2184 of the European Parliament and the Council of 16 December 2020 on the quality of water intended for human consumption—annex I part B (the minimum requirements for parametric values used to assess the quality of water intended for human consumption), the maximum allowed concentration of chromium in drinking water is set at 0.05 mg L^−1^ [2]. However, a parametric value of chromium 0.025 mg L^−1^ will be implemented, at least by January 2036 [3,4].

Therefore, the regulations and standards for water safety and protection result in the need to search for effective and economically justified water treatment unit processes. The available conventional methods for chromium decontamination from aqueous solutions can be categorized into several groups [5]. These include chemical precipitation (mostly in the case of the removal of chromates from chromate-rich wastewater, involving the reduction of Cr(VI) to Cr(III) at a high pH and its precipitation as chromium hydroxide or chromate-iron hydroxide, and then the utilization of the dewatered sludge), e.g., coagulation–precipitation–filtration, membrane technologies (reverse osmosis and nanofiltration, with an efficiency of 82–97%) [6,7], electrocoagulation, electrodialysis, reduction or ion exchange processes [8], and adsorption by iron oxides (ferrihydrite and goethite) and iron-oxide-coated sand (the removal of both Cr(III) and Cr(VI) requires pH changes). UV/TiO_2_ photocatalysis involves the reduction of Cr(VI) to Cr(III) in an acidic environment under the influence of a photocatalyst, anatase (TiO_2_), in the presence of Fe(III) ions. The use of ZnS as a photocatalyst provides further reduction of Cr(III) to metallic chromium [9]. Another method for chromium decontamination is based on a microbiological process using bacteria for reducing Cr(VI) to Cr(III), which is precipitated within biomass. Nevertheless, this method requires anaerobic conditions for effective Cr(VI) removal, thus, it may not be suitable for drinking water treatment [10,11]. Other methods for Cr(VI) removal involve nanomaterials based on, e.g., carbon nanotubes, graphene derivatives, layered double hydroxides (LDHs), and molybdenum disulfide (MoS_2_), etc. [12]. However, even the most advanced water treatment procedures have certain disadvantages and limitations, including sensitive processing conditions, a stern technological regime, and finally, further disposal and by-product problems, which can be expensive and unavoidable. Thus, in practice, very promising routes for cost-effective alternatives for water treatment processes based on the sorption phenomenon using natural aluminosilicates, called zeolites, are very often exploited due to their widespread abundance in nature [13,14].

**Figure 1:**
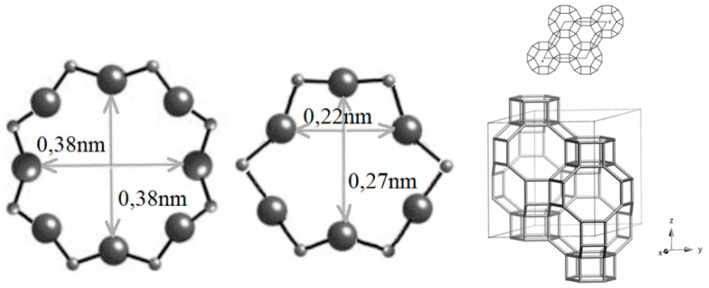
Chabazite framework structures [17].

Taking all the above into account, the authors have considered it of great interest to study and assess the capabilities of a natural low-cost and commonly available tectosilicate mineral—zeolite, namely chabazite (CH). Chabazite, as a crystalline microporous mineral, is predominantly composed of aluminosilicates, hence, it shows significant sorption properties. Chabazite belongs to the chabazite group, with a simplified formula (Na_2_,K_2_,Ca)_2_ [A_l4_Si_8_O_24_]·12 H_2_O. Chabazite’s framework structure comprises six eight-member rings of 0.38 × 0.38 nm, opening into large ellipsoidal cavities of 0.67 nm × 1.0 nm (chabazite cage) [15] or 0.67 nm × 1.1 nm [16,17,18], as shown in Figure 1.

The tetrahedral framework of chabazite, composed of SiO_4_ and AlO_4_ units, possesses a negatively charged surface, resulting in the ability to easily bind cations [19]. Therefore, zeolites are usually used to soften water (Ca^2+^ and Mg^2+^) and remove ammonium ions (NH_4_^+^), petroleum substances, radioactive elements, cationic heavy metals, and mycotoxins, viruses, and microorganisms. However, the anion-sorption properties of natural zeolites are limited [20,21]. Yet, the zeolite negative charge can be changed by surface chemical modification (SMZs—the surface modified zeolites) using surfactants, e.g., hexadecyltrimethylammonium bromide (HDTMA-Br and C_19_H_42_N^+^), to enhance its capability to remove anions from aqueous solutions. Thus, its surface modification is fully justified. Due to the bigger size of the HDTMA-Br molecules in relation to chabazite pore sizes, the modification of chabazite involves the sorption of the surfactant (based on cation exchange and hydrophobic bonding) only on the external surface of the zeolite, thus, HDTMA molecules are unable to penetrate the internal porous structure of chabazite [22]. However, in practice, surface modification never achieves perfect coverage on zeolite surfaces, therefore, SMZs are able to remove anions and also cations and non-polar organics [23,24]. Hence, SMZs have the potential to purify water from differently charged pollutants and for use in soil remediation applications.

The aim of this study was to determine the sorption properties of HDTMA-Br-modified chabazite towards Cr(VI) inorganic anions, including experimental research and theoretical investigation on sorption isotherms and kinetics, in order to explore the Cr(VI) binding mechanism. Prior to adsorption studies, raw chabazite was effectively modified in order to obtain double-layer surface coverage, defined as double external cation exchange capacity, under closed-circuit flow conditions. Subsequently, experimental studies on adsorption were conducted under batch conditions. The correct understanding and interpretation of adsorption isotherms is crucial for an overall improvement in adsorption mechanisms and the effective design of adsorption systems [25,26]. Therefore, the process equilibrium was described by 11 selected isotherm equations, while, to describe the sorption kinetics on the solution–solid interface, reaction models (reversible pseudo-first-order, reversible pseudo-second-order, irreversible pseudo-first-order, and irreversible pseudo-second-order) were used. Based on the mathematical optimization of the isotherms’ model parameters and kinetics models (performed by the means of a non-linear regression method based on the Levenberg–Marquardt algorithm [27] applied to minimize the sum of the squares of the error (SSE) function), a peculiar and original dual approach to the interpretation of the Cr(VI) adsorption mechanism was proposed. Furthermore, the overriding goal of this research was to obtain promising and effective material for water treatment applications dedicated to toxic Cr(VI) oxyanion removal from aqueous solutions.

## 2. Materials and Methods

### 2.1. Chabazite Characteristics

The high-purity natural chabazite used in this research was provided by St. Cloud Mining Company, originating from natural deposits in Bowie, MD, USA. The chemical composition of raw zeolite (reported as % *w*/*w* content of mineral oxides) was determined as follows: SiO_2_ 58.617%, CaO 2.518%, Al_2_O_3_ 14.549%, MgO 0.827, Fe_2_O_3_ 3.245%, Na_2_O 5.052%, K_2_O 1.253%, TiO_2_ 0.187%, SrO 0.129%, and ThO_2_ 0.143%, using X-ray fluorescence spectrometry (XRF, Axios.max, PANalytical, Almelo, The Netherlands). Its mineralogical composition was as follows: 54% chabazite, 36% clinoptilolite, 5% quartz, and 5% unidentified, which was determined on an X-ray diffractometer (XRPD, KRISTALLOFLEX 4H, Siemens, Munich, Germany). The Si/Al ratio (3.56) was determined based on the chemical composition of raw chabazite and confirmed the presence of a crystalline structure in the studied material. The identified order of exchangeable cations resulting from the material’s chemical composition was established as follows: Na^+^ > Fe^2+^ > Ca^2+^ > K^+^ > Mg^2+^ > Th^4+^. However, the purchased zeolite mineral was in the form of granulate (coarse powder), so further additional milling processing was required to ensure a uniform size distribution. To achieve this, a vibrational ball mill (vibratory grinder LMW-S TESTCHEM, Radlin, Poland) was used to obtain proper fine particles. Then, chabazite was sieved using a precise laboratory screen (Haver & Boecker, Münster, Germany) to collect a grain size ranging from 0.2 to 0.5 mm. Next, the separated fraction was repeatedly rinsed using demineralized water (RO with ultra-low-pressure membrane of 2.84 m^3^d^−1^, Vontron Technology Co., Guizhou, China) to eliminate any dusty fraction left after the grinding process, and it was finally dried at 110 °C in laboratory dryer (SLW 53 STD, POL EKO, Wodzislaw Slaski, Poland) for 24 h [28]. Textural parameters, such as the specific surface area (*A*_BET_) and total pore volume (*V*_T_), were determined by the N_2_-BET method using the Surface Area Analyzer (ASAP 2420, Micromeritics, Norcross, GA, USA) based on the Brunauer–Emmett–Teller theory. The measurement was conducted at a liquid nitrogen temperature of −196 °C, right after degassing the sample at 22 °C. The external surface area (*A*_ext_) and micropore surface area (*A*_mic_) were determined using De Boer’s t-plot method [29]. The obtained values for the textural parameters are presented in Table 1.

The surface morphology of the raw, activated, and modified chabazite samples were examined using scanning electron microscopy (VEGA3, TESCAN, Brno, Czech Republic)obtaining micrographs at magnifications of ×5000 and ×10,000 and bar scales of 10 and 5 µm. Before measurement, the samples were sputtered with silver in a low-vacuum sputter (Q150R S/E/SE, Quorum, East Sussex, UK).

All chemicals used in this study were of analytical grade.

### 2.2. Chabazite Pre-Treatment and Modification

#### 2.2.1. Chabazite Activation

Before chabazite surface modification, the zeolite was pre-treated (activated) in order to obtain the maximum sorption capacity. The activation step consisted of preparing the homo-ionic form (Na) of the zeolite. In this regard, 50 g of chabazite was contacted with 0.25 L of 0.5 M sodium chloride solution (NaCl 0.99, Merck, Rahway, NJ, USA) and placed in an orbital mechanic shaker for 24 h at 100 rpm (WElectronic, WL2000, Warsaw, Poland). Next, the sample was left for 3 h for zeolite sedimentation and then sodium chloride solution was decanted to separate the liquid from the solid phase. The excess of chlorides present on the chabazite grains’ surfaces was removed using high-retention seamless cellulose tubing (D9402-1FT Sigma Aldrich, St. Louis, MO, USA) until the chloride concentration left in the solution reached <1.0 mg L^−1^. The Cl^−^ ion concentration was measured by complexometric titration according to the international standard ISO 9297:1989 using the Mohr method [30]. Finally, the Na form of chabazite (CH-Na) was dried for 24 h at a constant temperature of 105 °C [28].

#### 2.2.2. CEC and ECEC Determination for Raw Chabazite

The chabazite cation exchange capacity (CEC) was determined based on ion exchange reactions (Na^+^/Ca^2+^). The Na form of the zeolite was contacted with calcium chloride solution (Merck) until the Ca^2+^ ions were exchanged with the Na^+^ ions present in the chabazite after activation (described above). Briefly, a sample of 10 g of the Na form of chabazite was placed in 1 M CaCl_2_ solution for 24 h. Afterwards, the Na^+^ content in the liquid phase was analyzed by a spectro-analytical procedure using atomic absorption spectroscopy AAS (SpectrAA 880, Varian, Santa Clara, CA, USA) at a wavelength of 586.6 nm (Table 2).

The external cation exchange capacity (ECEC) was determined by three independent methods in order to confirm the accuracy of the obtained results (Table 2).

The first method (I) was conducted according to Bardon’s procedure [31,32]. The method involved the use of hexaamminecobalt(III) chloride (Co(NH_3_)_6_Cl_3_, Merck). In brief, a sample of 1.0 g of chabazite (CH-Na) was placed in 25 mL of hexaamminecobalt(III) chloride solution (concentration of 1.0 g_Co(NH3)6Cl3_ L^−1^) for 24 h. Then, the liquid phase was filtered using syringe filters equipped with mixed cellulose ester (MCE, 0.2 µm Whatman) and spectrometrically analyzed for hexaamminecobalt(III) concentration using UV–VIS DR 5000 (HACH LANGE, Düsseldorf, Germany) [33]. The ECEC value was calculated based on the molar balance (Table 2).

The second method (II) involved the adsorption of tetraethylammonium bromide (TMA, C_4_H_12_N^+^, 0.98, Sigma-Aldrich) on the Na form of chabazite. In order to carry this out, 1.0 g of the Na form of CH was contacted and mixed using a hot-plate magnetic stirrer with 100 mL of demineralized water at a constant temperature of 80 °C. Next, portions of 10 mL of 1% TMA solution were added for every 1.5 h period of time, until the total solution volume reached 200 mL. Then, in the decanted solution, the total organic carbon (TOC) concentration was measured using a GE Analytical Instrument (TOC Sievers InnovOx, Trevose, PA, USA). The amount of surfactant left in the liquid phase, corresponding to the TMA equilibrium concentration in the sorbent (solid phase), was calculated from the mass balance equation (Equation (1)) and is shown in Table 2.

𝑞_eTMA_ = 𝑉_TMA_·(𝐶_0 TMA_ − 𝐶_e TMA_/𝑚_CH_) (1)

where *C*_0 TMA_ and *C*_e TMA_ are the initial and equilibrium TMA concentrations in the solution (g L^−1^), respectively, *V* is the volume of TMA solution (L), and *m*_CH_ is the sorbent dosage (g L^−1^).

The third method (III) used was CHN elemental analysis (CHNS, Vario EL III, Elementar, Hanau, Germany). After TMA adsorption, a chabazite sample was washed using hot water (80 °C) until Br^−^ ions were removed. The determination of the Br^-^ ion concentration was performed by complexometric titration according to the international standard ISO 9297:1989 using the Mohr method [30]. Afterwards, chabazite was washed again using hot ethanol (96%), then dried at 105 °C, and the CHN elemental concentration in the solid phase was analyzed [34]. The ECEC was determined from the difference between the CHN contents of the raw chabazite and the TMA-Br-treated material.

The obtained results shown in Table 2 are given as mean values from three repeated measurements. The replicability of the results obtained for the ECEC clearly indicates correctly performed determinations using three independent determination methodologies, and, thus, provides confidence that the ECEC is 0.262 mmol g^−1^.

#### 2.2.3. Chabazite HDTMA-Br Modification

The Na form of chabazite was modified by HDTMA-Br surfactant solution (Mw: 364.4 g mol^−1^, Merck) in the concentration corresponding to 2ECEC, resulting in double-layer chabazite surface coverage (calculated from Equation (2)).

𝑚 _HDTMA_ = (𝑀/𝑃) · 2ECEC · 𝑚_CH_ (2)

where *m*_CH_—chabazite sample mass (g) and *M* and *P*—HDTMA-Br molar mass (g mmol^−1^) and purity (0.98) of HDTMA-Br, respectively.

**Figure 2:**
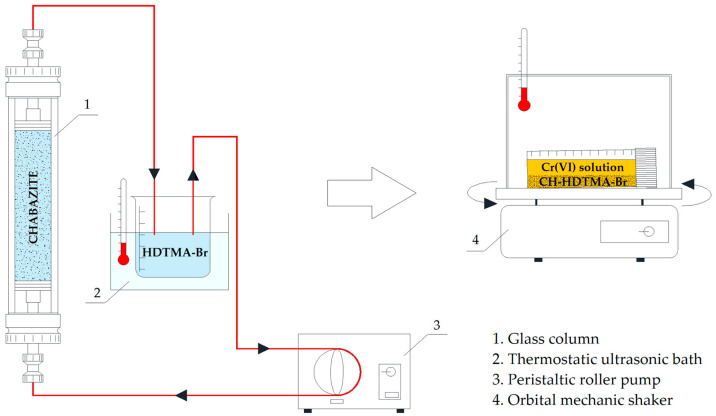
Scheme of analyzed system for chabazite surface modification under closed-circuit flow conditions (**left**) and Cr(VI) adsorption under batch conditions (**right**).

The HDTMA-Br solutions were prepared by dissolving the surfactant in demineralized water to obtain an initial concentration of *C*_0 HDTMA_ = 1050 mg_HDTMA_ L^−1^ (intentionally, the surfactant concentration was above the critical micelles concentration (CMC) in order to obtain 2ECEC and was conducted in a thermostatic ultrasonic bath (Sonic-2, Polsonic, Warsaw, Poland) at 40 °C to avoid surfactant solution crystallization). The modification was carried out under closed-circuit conditions using a column made of glass, 10 cm high with a 10 mm inner diameter. The glass column was filled with a CH-Na zeolite bed 5 cm in height with a chabazite mass of 3.5 g per bed (Figure 2). The column was fed by HDTMA-Br solution from the bottom to the top at a constant flow rate (4 mL min^−1^) using a peristaltic roller pump.The chabazite modification process was conducted until the concentration of HDTMA-Br outlet solution (*C*_out HDTMA_) was constant.

The degree of surface coverage (*DC*, multiples of ECEC) was calculated from the following equation (Equation (3)):

𝐷𝐶 = 𝑥 · (𝐶_0 HDTMA_ − 𝐶_𝑜𝑢𝑡 HDTMA_)/𝐶_0 HDTMA_ (3)

where *x* = 2 (meaning degree of coverage), *C*_out HDTMA_ is the surfactant concentration in the column outlet (mg L^−1^), and *C*_0 HDTMA_ is the initial surfactant concentration *C*_0 HDTMA_ = 1050 mg _HDTMA_ L^−1^.

After modification, the bed (surfactant-modified zeolite—SMZs) was removed from the column and the excess of HDTMA-Br was repeatedly rinsed with demineralized water to eliminate Br^−^ ions until they were not detected (the Mohr method). Changes in the HDTMA-Br solution concentrations were determined using a TOC analyzer before zeolite was applied for research use. Then, the HDMTA-Br concentration was calculated as the difference between its initial concentration and its concentration in the solution after the modification process. Finally, HDTMA-Br-modified chabazite (CH-HDTMA-Br) was dried at a constant temperature of 105 °C for 24 h.

### 2.3. Experimental Methods—Cr(VI) Sorption on CH-HDTMA-Br

#### 2.3.1. Equilibrium Studies

Chromium adsorption studies were performed under batch conditions (Figure 2). In total, 33 samples of 0.1 g of CH-HDTMA-Br were incubated for 24 h at room temperature in 10 mL of initial solutions under stirring conditions (140 rpm) using an orbital mechanic shaker (WElectronic, WL2000, Warsaw, Poland). The initial solutions’ concentration (*C*_0_) of Cr(VI) ions ranged from 0.9 to 1700 mg_Cr(VI)_ L^−1^. They were prepared by the dilution of stock solution based on potassium chromate (K_2_CrO_4_, Sigma-Aldrich). After 24 h of the sorption process, the equilibrium adsorbate concentrations in the liquid phase (*C*_e_) were determined for each sample. To achieve this, inductively coupled plasma-optical emission spectroscopy (ICP-IOS) was used according to the EN ISO 11885:2007 [35] standard using the Integra XL apparatus (GBC Scientific Equipment, Perai, Malaysia). The values of the chromium equilibrium concentration were determined at a wavelength of 396.847 nm and calculated as a mean value from three measurements. The detection limit was 2 µg_Cr_ L^−1^ and the R^2^ for the standard curve was 0.999. Finally, all the described above steps allowed for the determination of the chromium adsorption isotherm..obtained isotherm illustrates the relationship between the amount of adsorbed adsorbate (solid phase) per unit mass of the adsorbent (*q*_e_), calculated from the following mass balance equation (Equation (4)) and the equilibrium concentration of the adsorbent (*C*_e_).

𝑞_e_ = 𝑉·(𝐶_0_ − 𝐶_e_/𝑚_CH_) (4)

where *q*_e_—equilibrium concentration of Cr(VI) in the sorbent (solid phase), (mg g^−1^); *C*_0_—initial adsorbate (Cr(VI)) concentration in the liquid phase, (mg L^−1^); *C*_e_—equilibrium adsorbate Cr(VI) concentrations in the solution, (mg L^−1^); *V*—volume of Cr(VI) solution, (L); and *m*_CH_—mass of sorbent used, (g).

The results of the equilibrium experimental studies, such as *C*_e_, *C*_0_, and *q*_e_ at different initial Cr(VI) concentrations, are shown in the Table 3.

#### 2.3.2. Kinetics Studies

In order to describe the sorption kinetics of Cr(VI) ions onto CH-HDTMA-Br, the adsorbent sample (0.1 g) was placed in chromium-containing solutions (10 mL) with the following initial Cr(VI) concentrations (*C*_0_): 104.00; 154.13; 194.00; 217.49; 281.24; and 309.75 mg L^−1^ for 1; 3; 5; 10; 20; 30; 60; 90; 120; 180; 240; 303; 600; and 1440 min under batch conditions. At the established contact times, the chromium content (*C*_e_) in the solutions was determined, while the concentration of Cr(VI) ions in the solid phase (*q*_e_) was calculated from the mass balance equation (Equation (2)). Similarly, the Cr(VI) concentration was measured using an ICP-OES analyzer, according to the procedure described above.

### 2.4. Morphological Study

**Figure 3:**
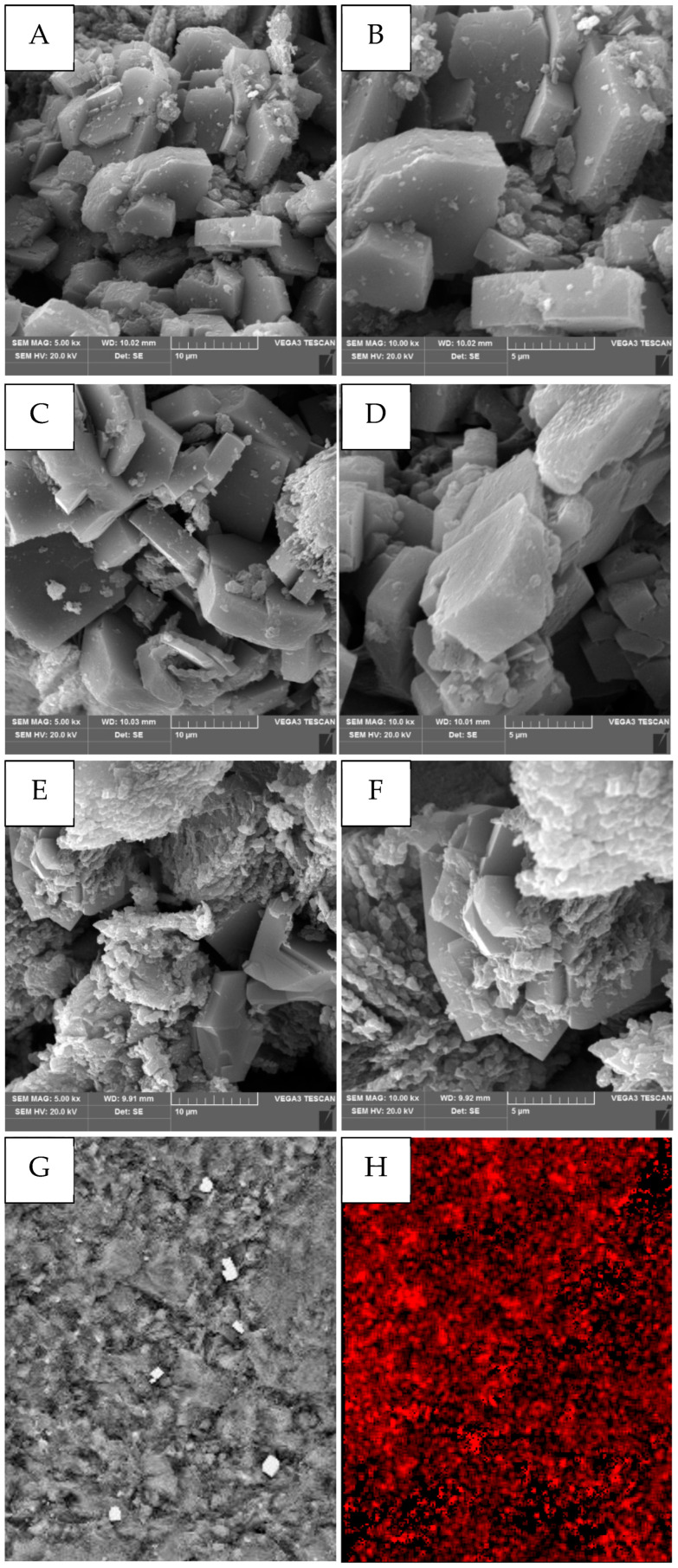
Scanning electron micrographs: (**A**,**B**)—raw samples, images (**C**,**D**)—activated samples, images (**E**,**F**)—modified samples (at magnification ×5000 and ×10,000 and the bar scale 10 and 5 µm), images (**G**,**H**)—the EDS analyse of chabazite after modification: mapping of carbon distribution (right), map dimension 85 µm × 67 µm (×1500).

The SEM images shown in Figure 3 expose noticeable changes in the morphological structure of exanimated chabazite samples (images A and B represent raw samples; images C and D represent activated samples; and images E and F represent modified samples, respectively).

The amount of sorbed surfactant depended on the mineralogical composition and structural properties of the modified zeolite. These also determined the total cation exchange capacity CEC, external cation exchange capacity ECEC, specific surface area, and pore size. The highest amount of adsorbed HDTMA was determined for mordenite, and the smallest for clinoptilolite. Different results were obtained regarding structure, specifically regarding the diameters of the sorption channels. If the modifier molecules were too large to penetrate the zeolite pores, their sorption was limited to the external surface of the zeolite and was converted into external cation exchange capacity ECEC [36,37]. ECEC could constitute 10–50% of the total ion exchange capacity of the zeolite [38].

The micrographs (Figure 3) of raw chabazite, as presented in images A and B, show crystals arranged in disks and elongated chains consisting of small (from 0.2 to 1.0 μm), well-developed rhombohedra. Additionally, individual and grouped (from 1 μm to 15 μm) hexagonal crystals with a tabular shape can be observed. Images of activated chabazite, as presented in micrographs C and D, indicate a lack of tangible differences between them in comparison to raw samples (A and B). The obtained results presented in micrographs E and F reveal a noticeable effect of modification on the chabazite surface. The sharp edges of the zeolite crystals visible in the SEM micrographs A–D are invisible in the SEM micrographs of HDTMA-modified materials (E and F). The modified samples reveal an extensive irregular external surface, with agglomerates of spherically shaped particles, slightly resembling a spongy structure, which suggests that the exanimated samples indicated a complex and extended surface area after chabazite HDTMA-Br modification.

The HDTMA-Br molecule has a head diameter that is too large (0.694 nm [39]) to penetrate deep into the zeolite structure (the sorption channel diameters are too small). Therefore, a change in electrostatic charge only affects its external surface [40,41]. According to the authors of [42], modified zeolites have a smaller specific surface area because the upper entrance part to the channels is blocked by the surfactant. The consequence of this results in a significant limitation of cation diffusion into the interior of the zeolite structure. Since surfactant sorption occurs on the external surface, the process does not affect the connections of the Si-O and Al-O oxygen bridges of the structure. However, it has been found that, as a result of such modification, the interlayer water content decreases, which is caused by the hydrophobization of the surface and the substitution of water molecules by HDTMA-Br cations [43,44,45].

Moreover, EDS analysis (Quanta 3D FEG, FEI Hillsboro, OR, USA) confirmed the presence of HDTMA-Br on the modified chabazite surface, based on the mapping of carbon distribution, as shown in Figure 3G,H.

The results of CHN analysis (CHNS Vario EL III, Elementar, Hanau, Germany), as presented in Table 4, additionally confirm the effective modification of the chabazite surface, resulting in an increase in C mg/100 g from 47 to the value of 754.5. Thus, this verifies the presence of HDTMA-Br on the chabazite surface.

## 3. Results and Discussion

### 3.1. Equilibria Modelling Part

Experimentally determined adsorption isotherms were used to describe the equilibrium of the sorption process. In this study, eleven isotherm models were involved to illustrate the relationship between the amount of adsorbate (chromium concentrations) adsorbed per adsorbent unit mass (CH-HDTMA-Br) and the equilibrium concentration of the adsorbent. Based on the experimentally obtained data shown in Table 3, the mathematical optimization of the isotherms’ model parameters was performed by the means of a non-linear regression method based on the Levenberg–Marquardt algorithm [27], applied to minimize the sum of the squares of the error (SSE) function (Equation (5)).

(5) $$\sum_{i=1}^{N}{\left(q_{e}-q_{e\;mod}\right)}_{i}^{2}=min$$

where *N*—number of experimental points; *q*_e_—equilibrium concentration of Cr(VI) in the sorbent (solid phase) determined experimentally, (mg g^−1^); and *q*_e mod_—equilibrium concentration of Cr(VI) in the sorbent calculated from a model (Equations (12)–(22)), (mg g^−1^).

All isotherm modeling calculations were implemented using the high-performance mathematical and analytical software tool Maple 16.2 (Maplesoft^TM^, Waterloo, ON, Canada). In order to avoid errors resulting from the linearization of the isotherm equation form, in this study, equilibria modeling was performed by implementing untransformed forms of non-linear equations (two-, three-, and four-parameter adsorption isotherms were implemented), which allowed for preserving the integrity of the obtained data [46,47,48].

**Figure 4:**
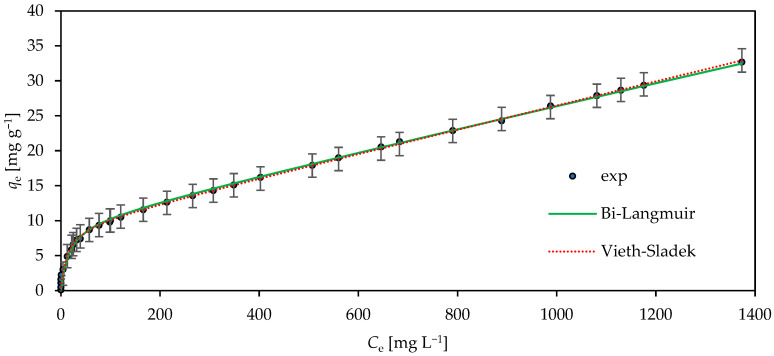
The Bi-Langmuir and Vieth–Sladek non-linear adsorption isotherm plot of Cr(VI) onto CH-HDTMA-Br.

The obtained estimated values of the isotherm parameters, such as the equilibrium constant (*K*), adsorption capacity (*q*_e mod_), and constant determining the heterogeneity (*n*, *n*_1_, *n*_2_, and *m*) of the CH-HDTMA-Br surface (refer to the equations in which these parameters occur), are shown in Table 5, and their visual interpretations as isotherm curve fittings are shown in Figure 4.

To enable the optimization procedure, the selected optimization standards were involved (non-linear error functions), therefore, the estimated parameters constitute the basis for selecting the most appropriate and best-fitted model for the experimental points.

The six non-linear error functions (Equations (6)–(11)) employed in this study are as follows: Fisher test (*F*); approximation of the standard deviation (*σ*); mean error (*ME*); coefficient of determination (*R*^2^); sum of squares due to error (*SSE*); and adjusted coefficient of determination (*Adj R*^2^) [49].

(6) $$F=\frac{\left(N-l\right)\sum_{i=1}^{N}{\left(q_{e,i}-\frac{1}{N}\sum_{i=1}^{N}q_{e,i}\right)}^{2}}{\left(N-1\right)\sum_{i=1}^{N}{\left(q_{e,i}-q_{mod,i}\right)}^{2}}\;$$

(7) $$\sigma =\sqrt{\frac{1}{N-l}\sum_{i=1}^{N}{\left(q_{e,i}-q_{mod,i}\right)}^{2}}$$

(8) $$ME=\frac{1}{N}\sum_{i=1}^{N}\left|\frac{q_{e,i}-q_{mod,i}}{q_{e,i}}\right|$$

(9) $$R^{2}=\frac{\sum_{i=1}^{N}{\left(q_{mod,i}-\frac{1}{N}\sum_{i=1}^{N}q_{e,i}\right)}^{2}}{\sum_{i=1}^{N}{\left(q_{e,i}-\frac{1}{N}\sum_{i=1}^{N}q_{e,i}\right)}^{2}}\;$$

(10) $$SSE=\sum_{i=1}^{N}{\left(q_{e,i}-q_{mod,i}\right)}^{2}\;$$

(11) $${Adj\;R}^{2}=1-\left(1-R^{2}\right)\frac{N-1}{N-l-1}=1-\left(1-\frac{\sum_{i=1}^{N}{\left(q_{mod,i}-\frac{1}{N}\sum_{i=1}^{N}q_{e,i}\right)}^{2}}{\sum_{i=1}^{N}{\left(q_{e,i}-\frac{1}{N}\sum_{i=1}^{N}q_{e,i}\right)}^{2}}\right)\frac{N-1}{N-l-1}$$

All calculated empirical model parameters (Equations (12)–(22)) and the determined error functions (Equations (6)–(11)) are tabulated in Table 5. Figure 4 shows plots comparing the selected theoretical Bi-L and V-S isotherm equations with experimental data (points).

**Table 5:** Equilibrium parameter values calculated using non-linear form of isotherm equations (Equations (12)–(22)).

Isotherm Abbreviations	Equation	Equation Number	*K* (L mg^−1^)	*q*_e mod_ (mg g^−1^)	Heterogeneity Parameter	*F*	*ME*	*σ*	*SSE*	*R* ^2^	*Adj R* ^2^
L	$q_{e}=\frac{q_{m}KC_{e}}{1+KC_{e}}$	(12)	0.002917	34.87	-	13.06	0.36	2.59	208.90	0.9258	0.9234
F	$q_{e}=KC_{e}^{\frac{1}{n}}$	(13)	1.244	-	n = 2.266	68.75	0.21	1.13	39.68	0.9859	0.9855
T	qe=qmKCe1+KCen1n	(14)	0.07627	27,950.0	n = 0.077	51.15	0.22	1.30	51.15	0.9818	0.9806
R-Pet	$q_{e}=\frac{q_{m}KC_{e}}{1+KC_{e}^{n}}$	(15)	779.9	0.065	n = 0.557	66.04	0.21	1.15	39.97	0.9858	0.9849
D-R	$\log q_{e}=-n\left(\log^{2}\left(K\cdot C_{e}\right)\right)+\log q_{m}$	(16)	3.24 × 10^−8^	129.50	n = 0.035	26.35	0.21	1.82	100.17	0.9644	0.9621
R-Pr	$q_{e}=\frac{q_{m}KC_{e}}{{\left(1+KC_{e}\right)}^{n}}$	(17)	126.4	0.142	n = 0.556	66.14	0.21	1.15	39.75	0.9859	0.9849
F-S	$q_{e}=\frac{q_{m}KC_{e}^{n_{1}}}{1+KC_{e}^{n_{2}}}$	(18)	17,600.0	14,900.0	n_1_ = 1.0 n_2_ = 0.5	46.31	0.24	1.37	55.10	0.9804	0.9784
L-F	$q_{e}=\frac{q_{m}{\left(KC_{e}\right)}^{n}}{1+{\left(KC_{e}\right)}^{n}}$	(19)	1.23 × 10^−7^	1.11 × 10^3^	n = 0.41	56.76	0.19	1.24	46.51	0.9835	0.9824
M-J	$q_{e}=\frac{q_{m}{\left(KC_{e}\right)}^{m}}{1+{\left(KC_{e}\right)}^{n}}$	(20)	2330	0.104	m = 0.778 n = 0.328	59.70	0.22	1.21	42.75	0.9848	0.9832
Bi-L	$q_{e}=\frac{q_{m,1}K_{1}C_{e}}{1+K_{1}C_{e}}+\frac{q_{m,2}K_{2}C_{e}}{1+K_{2}C_{e}}$	(21)	K_1_ = 1.77 × 10^−5^ K_2_ = 0.062	q_m1_ = 957.5 q_m2_ = 9.896	-	276.6	0.16	0.56	9.22	0.9967	0.9964
V-S	$q_{e}=K\;Ce+\frac{q_{m}\;n\;C_{e}}{1+nC_{e}}$	(22)	0.0179	9.399	n = 0.077	320.0	0.15	0.52	8.25	0.9971	0.9969

Where: *K*–equilibrium constant (L mg^−1^); *q*_e mod_—equilibrium concentration of Cr(VI) in the sorbent calculated from model (mg g^−1^); *n* and *m*—constant parameters determining heterogeneity of sorbent surface (dimensionless); *F*—Fisher test; *σ*—approximation of the standard deviation; *ME*—mean error; *R*^2^—coefficient of determination; *Adj R*^2^—adjusted coefficient of determination. Isotherms name abbreviation: L—Langmuir; F—Freundlich; T—Toth; R-Pet—Redlich–Peterson; D-R—Dubinin–Radushkevich; R-Pr—Radke–Praushnitz; F-S—Fritz–Schlunder; L-F—Langmuir–Freundlich; M-J—Marczewski–Jaroniec; Bi-L—Bi-Langmuir; V-S—Vieth–Sladek.

Particularly noteworthy are the Bi-Langmuir and Vieth–Sladek isotherms, which are characterized by an excellent agreement with the obtained experimental data (*Adj R*^2^ above 0.996), as shown in Figure 4. Both mathematical models are based on the assumption that the retention of the active solution component consists of at least two mechanisms. The Bi-Langmuir isotherm approach assumes that, during adsorption, the solute is retained on an energetically heterogeneous surface of the sorbent with two types of independent binding sites, and the nature of binding at both sites can be described by a separate Langmuir equation. As a result, the isotherm model consists of the sum of two additive terms, which are Langmuir equations [50,51,52]. Due to the fact that the value of sorption capacity *q*_1_ is very high (*q*_m1_ = 957.5 mg g^−1^) and the equilibrium constant *K*_1_ is very small (*K*_1_ = 1.77 × 10^−5^ L mg^−1^), it should be assumed that, in the case of this type of active site (index 1 in model equation), adsorption is almost linear (directly proportional to the concentration in the liquid phase *q*_e_ ∝ *K*_1_) and is not limited by the number of active sites. Moreover, sorption involving the second type of active site (index 2 in model equation) is typical for non-linear isotherms growing asymptotically to the value determined by the maximum sorption capacity (*q*_m2_ = 9.896 mg g^−1^). In the case of the Vieth–Sladek model, retention is defined as transient adsorption, which includes regular surface adsorption (non-linear component) and the trapping of the active ingredient in capillaries of the amorphous structure within the sorbent (linear component) [53,54]. Interestingly, the values of the maximum sorption capacity in both models reached very similar values (*q*_2_ = 9.896 mg g^−1^ in the Bi-Langmuir equation and *q*_e mod_ = 9.399 mg g^−1^ in the Vieth–Sladek equation). Therefore, the following interpretation of the obtained results can be proposed. The retention of chromium (VI) ions in the porous internal surface of chabazite is a process that consists of surface adsorption taking place on one type of active site according to the Langmuir mechanism, with the maximum adsorption capacity of the material (CH-HDTMA-Br) being approximately 9.3–9.9 mg g^−1^, and the trapping of chromium ions in the capillaries of the amorphous sorbent, proportional to the concentration of chromium ions in a liquid phase and independent of the number of active sites on the chabazite surface in the studied concentration range. Such a retention mechanism may be an explanation for the pronounced growth of the Cr(VI) ion concentration in the adsorbed phase (*q*_e_) versus the Cr(VI) ion concentration in the liquid phase (*C*_e_), without reaching a distinct plateau, characteristic for sorbents with a sorption capacity controlled by a limited number of active sites on the surface.

Moreover, other error functions (Table 5) were also employed in this study to assess the goodness of fit of the model isotherm equations to the experimental equilibrium data. All the applied error statistics gained the most favorable values for both isotherms mentioned, namely, *F* test (Bi-L *F* = 276.64 and V-S *F* = 320.01), approximation of the standard deviation (Bi-L *σ* = 0.56; V-S *σ* = 0.52), mean error (Bi-L *ME* = 0.16; V-S *ME* = 0.15), and sum of squares error (Bi-L *SSE* = 9.22; V-S *SSE* = 8.25).

### 3.2. Kinetics Modelling Part

In this study, the expressions originally given by Lagergren were used to describe the theoretical adsorption kinetics on the liquid–solid interface [55,56]. The models’ parameters were fitted based on a non-linear fitting method using software tool Maple 16.2. The appropriate values of *C*_0_, *C*_t_, and m_CH_ were used to calculate the instantaneous concentration of chromium (*q*_t_) required further for model calculations of adsorption kinetics (Equation (23), Table 6).

𝑞_t_ = 𝑉·(𝐶_0 —_ 𝐶_t_/𝑚_CH_) (23)

where *q*_t_—calculated values of instantaneous concentration of chromium ions in the adsorbed phase (mg g^−1^); *C*_0_—initial adsorbate Cr(VI) concentration in the liquid phase (mg L^−1^); *C*_t_—adsorbate Cr(VI) instantaneous concentration in the liquid phase (mg g^−1^), and *m*_CH_—mass of a sample. Series number I-VI—the initial concentrations (*C*_0_) of chromium anions, 104.00; 154.13; 194.00; 217.49; 281.24; and 309.75 (mg L^−1^), respectively.

The selection of the most appropriate adsorption kinetics model among the reversible pseudo-first-order, the reversible pseudo-second-order, the irreversible pseudo-first-order, and the irreversible pseudo-second-order models (Equations (24)–(27)), as presented in Table 7, was performed. The constant rates (*k*_11_, *k*_21_, *k*_1_, and *k*_2_), as well as the maximum concentration *q*_m_, which are the kinetic models’ parameters, were calculated using the mentioned non-linear regression method based on the Levenberg–Marquardt algorithm. The obtained results supplemented by the appropriate statistical error functions are shown in Table 7.

**Figure 5:**
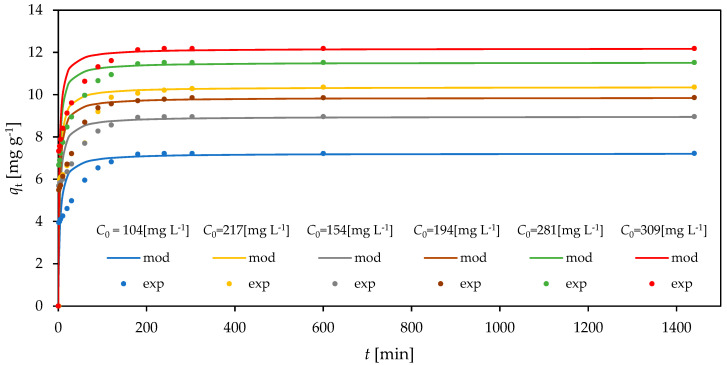
Time profiles of Cr(VI) adsorption onto CH-HDTMA-Br described by the irreversible pseudo-second-order model.

Based on the obtained results, the change in the Cr(VI) ion concentration in the adsorbent (*q*_t_) over time (*t*) for different initial concentrations in the solution can be most precisely described by the irreversible pseudo-second-order model (Figure 5, where the points represent experimental data). The applied error functions (the highest *F* value and the closest to one *R*^2^ and *Adj R*^2^ values) indicate the pseudo-second-order irreversible model (Equation (27), Table 7) as the most appropriate among the tested models to describe the studied process kinetics, which most probably indicate that the sorption mechanism involves the volumetric filling of pores at a simultaneously occurring chemical reaction.

## 4. Summary

Toxic chromium, due to its bioaccumulation and mutagenic properties, poses a particular threat to human health and the environment. In this study, the sorption of Cr(VI) onto HDTMA-Br-modified chabazite from aqueous solutions was studied. Experimentally obtained (under batch conditions) adsorption isotherms were used to describe the equilibrium of the sorption process. However, before adsorption studies, raw chabazite was activated to obtain a homo-ionic Na^+^ form of the zeolite, which was then modified by the immobilization of hexadecyltrimethylammonium bromide in order to change its adsorption abilities towards anions. The equilibria modeling was performed by implementing untransformed forms of non-linear equations (two-, three-, and four-parameter adsorption isotherms were used). The correlation of equilibrium data by either theoretical or empirical equations is essential to the practical design and operation of adsorption systems, hence, it is important to establish the most appropriate correlation for the equilibrium curves. Moreover, the correct understanding and interpretation of adsorption isotherms is crucial for overall improvements inadsorption mechanisms. Further, the adsorption equilibrium and kinetics studies provide an understanding of the adsorption mechanism, thus, the possibility of predicting and optimizing the sorption process based on mathematical modeling. Hence, the equilibria were described using Langmuir, Freundlich, Toth, Redlich–Peterson, Dubinin–Radushkevich, Radke–Praushnitz, Fritz–Schlunder, Langmuir–Freundlih, Marczewski–Jaroniec, Bi-Langmuir, and Vieth–Sladek isotherm equations. Among the analyzed adsorption models, the most accurate fit was achieved by the Vieth–Sladek and Bi-Langmuir isotherm equations, and the values of the maximum sorption capacity in both models (*q*_2_ = 9.896 mg g^−1^ in the Bi-Langmuir equation and *q*_e mod_ = 9.399 mg g^−1^ in the Vieth–Sadek equation) were very close and demonstrated a high degree of precision in predicting the Cr(VI) ion adsorption behavior on the CH-HDTMA-Br surface. The obtained results suggest that the studied adsorption process takes place according to both the Langmuir mechanism, with the maximum adsorption capacity of the modified chabazite reaching approximately 9.3–9.9 mg g^−1^, and simultaneously to the trapping of Cr(VI) in the capillaries of the amorphous sorbent. Therefore, such a retention mechanism may explain the significant increase in the Cr(VI) ion concentration in the adsorbed phase (*q*_e_) versus the Cr (VI) ion concentration in the liquid phase (*C*e), without reaching a distinct plateau. In order to describe the sorption kinetics on the solution–solid interface, the reaction models (including the pseudo-first- and second-order reversible and irreversible equations) were used. It was established that, among the examined models, the most appropriate one for description of the studied process kinetics was the pseudo-second-order irreversible model, which most probably indicates that the sorption mechanism involves the volumetric filling of pores at a simultaneously occurring chemical reaction. However, further studies and interpretation may be necessary to fully understand the relationship between the adsorbate and adsorbent in this context.

However, these results indicate that effective zeolite surface modification allows for changing its ability towards effective anion binding and may play an important role in current research directions for eco-effective and cheap adsorbents for water treatment applications. Therefore, the results have powerful implications for Cr(VI) removal on an industrial scale and the management of Cr(VI)-bearing water sites affected by the tanning and electroplating industry, which generates wastewater containing several hundred grams of chromium per liter.

In summary, the results obtained in this study provide a theoretical basis for the further development and application of chabazite as an effective adsorbent for Cr(VI) removal. Furthermore, such modified zeolite may become a promising adsorbent for other anionic contaminants present in aqueous environments. Overall, this research may be assigned to sustainable water treatment management practices using natural abundant zeolites as an effective and economically affordable Cr(VI) adsorbent.

## Figures and Tables

**Table 1:** Textural parameters of raw chabazite determined by N2 adsorption at temperature of −196 °C (* De Boer’s t-plot method).

Surface Area (m^2^ g^−1^)			Pore Volume (mL g^−1^)
*A*_BET_	*A*_mic_ *	*A*_ext_ *	*V*_T_
340.22	276.23	63.99	0.30

**Table 2:** Exchange capacity of chabazite.

Exchange Capacity	CEC (mmol g^−1^)	ECEC (mmol g^−1^)		
	Method AAS	Method I VIS	Method II TOC	Method III CHN
	1.6570	0.2625	0.2617	0.2621

**Table 3:** The results of the experimental studies.

***C*_0_**	**(mg L^−1^)**	**0.9**	**10.9**	**4.9**	**15.9**	**22.8**	**34.5**	**61.6**	**80.1**	**90.6**	**104.0**	**113.1**
***C*_e_**	**(mg L^−1^)**	0.0	0.0	0.0	0.0	0.6	4.3	12.9	21.6	25.0	31.8	39.1
***q*_e_**	**(mg g^−1^)**	0.1	1.1	0.5	1.6	2.2	3.0	4.9	5.9	6.6	7.2	7.4
***C*_0_**	**(mg L^−1^)**	**143.8**	**170.4**	**195.9**	**200.0**	**225.8**	**281.2**	**340.1**	**401.5**	**450.1**	**500.0**	**564.0**
***C*_e_**	**(mg L^−1^)**	57.4	77.0	98.7	100.0	120.8	166.1	213.8	265.8	307.4	348.7	402.5
***q*_e_**	**(mg g^−1^)**	8.7	9.3	9.8	10.0	10.5	11.6	12.6	13.6	14.3	15.1	16.2
***C*_0_**	**(mg L^−1^)**	**685.6**	**750.0**	**851.2**	**892.0**	**1019.1**	**1131.4**	**1251.6**	**1359.7**	**1415.5**	**1468.2**	**1700.0**
***C*_e_**	**(mg L^−1^)**	507.1	559.9	645.7	683.0	790.3	889.0	987.4	1080.9	1129.5	1175.7	1373.4
***q*_e_**	**(mg g^−1^)**	17.9	19.0	20.5	21.3	22.9	24.3	26.4	27.9	28.7	29.4	32.7

**Table 4:** Results of CHN analysis for chabazite after modification.

Zeolite	C [mg/100 g]	H [mg/100 g]	N [mg/100 g]	Suma CHN	q_eHDTMA_ [mg/g]
CH-Na	47.0	190.0	484.0	721.0	-
CH-HDTMA-Br	754.5	11,016.0	1857.0	13,627.5	165.31

**Table 6:** Kinetics study—experimental data.

Series Number		I	II	III	IV	V	VI
*t* (min)	*C*_0_ (mg L^−1^)	104.00	154.13	194.00	217.49	281.24	309.75
1	*q*_t_ (mg g^−1^)	3.95	5.69	5.49	5.99	6.67	7.33
	*C*_t_ (mg L^−1^)	64.50	97.21	139.10	157.60	214.54	236.44
3	*q*_t_ (mg g^−1^)	4.00	5.74	5.60	6.10	6.90	7.56
	*C*_t_ (mg L^−1^)	64.00	96.71	138.00	156.50	212.24	234.14
5	*q*_t_ (mg g^−1^)	4.11	5.85	5.71	6.21	7.23	7.89
	*C*_t_ (mg L^−1^)	62.90	95.61	136.90	155.40	208.94	230.84
10	*q*_t_ (mg g^−1^)	4.26	6.00	6.14	6.64	7.75	8.41
	*C*_t_ (mg L^−1^)	61.40	94.11	132.60	151.10	203.74	225.64
20	*q*_t_ (mg g^−1^)	4.61	6.35	6.71	7.21	8.47	9.13
	*C*_t_ (mg L^−1^)	57.90	90.61	126.90	145.40	196.54	218.44
30	*q*_t_ (mg g^−1^))	4.98	6.72	7.21	7.71	8.95	9.61
	*C*_t_ (mg L^−1^)	54.18	86.89	121.86	140.36	191.74	213.64
60	*q*_t_ (mg g^−1^)	5.96	7.70	8.70	9.20	9.97	10.63
	*C*_t_ (mg L^−1^)	44.43	77.14	107.00	125.50	181.54	203.44
90	*q*_t_ (mg g^−1^)	6.54	8.28	9.38	9.88	10.66	11.32
	*C*_t_ (mg L^−1^)	38.65	71.36	100.22	118.72	174.64	196.54
120	*q*_t_ (mg g^−1^)	6.82	8.57	9.57	10.07	10.95	11.61
	*C*_t_ (mg L^−1^)	35.76	68.47	98.30	116.80	171.74	193.64
180	*q*_t_ (mg g^−1^)	7.19	8.93	9.72	10.21	11.46	12.12
	*C*_t_ (mg L^−1^)	32.15	64.86	96.85	115.35	166.64	188.54
240	*q*_t_ (mg g^−1^)	7.22	8.96	9.79	10.29	11.52	12.18
	*C*_t_ (mg L^−1^)	31.81	64.52	96.13	114.63	166.02	187.92
303	*q*_t_ (mg g^−1^)	7.22	8.96	9.86	10.36	11.52	12.18
	*C*_t_ (mg L^−1^)	31.81	64.52	95.41	113.91	166.02	187.92
600	*q*_t_ (mg g^−1^)	7.22	8.96	9.86	10.36	11.52	12.18
	*C*_t_ (mg L^−1^)	31.80	64.51	95.41	113.91	166.01	187.91
1440	*q*_t_ (mg g^−1^)	7.22	8.96	9.86	10.36	11.52	12.19
	*C*_t_ (mg L^−1^)	31.79	64.50	95.40	113.90	166.00	187.90

**Table 7:** Non-linear (integral) equation forms, calculated kinetic parameters, and error functions for Cr(VI) adsorption onto CH-HDTMA-Br.

Kinetic Model Equations	No.	*C*_0_ (mg L^−1^)	*q*_e_ (mg g^−1^)	Model Parameters	*F* -	*R*^2^ -	*Adj R*^2^ -
Pseudo-first-order reversible $q_{t}=q_{e}\left(1-e^{-k_{11}\frac{q_{m}}{q_{e}}t}\right)$	(24)	104.00	7.221	$k_{11}$ = 1.9192 × 10^−1^ $q_{m}$ = 12.593 (mg g^−1^)	1.894	0.767	0.728
		154.13	8.963		2.343	0.698	0.648
		194.00	9.860		2.610	0.741	0.698
		217.49	10.359		2.682	0.719	0.672
		281.24	11.524		2.913	0.674	0.620
		309.75	12.185		2.802	0.650	0.592
Pseudo-second-order reversible $q_{t}=\frac{q_{m}q_{e}(e^{z_{22rq}k_{21}t}-1)}{q_{m}(1+e^{z_{22rq}k_{21}t})-2q_{e}}$ where: *z_22rq_ =* $\frac{2q_{m}\left(q_{m}-q_{e}\right)}{q_{e}}$	(25)	104.00	7.221	$k_{21}$ = 5.49 × 10^−3^ $q_{m}$ = 21.015 (g mg^−1^min^−1^)	1.743	0.767	0.728
		154.13	8.963		2.287	0.702	0.653
		194.00	9.860		2.560	0.743	0.700
		217.49	10.359		2.671	0.720	0.673
		281.24	11.524		2.974	0.672	0.617
		309.75	12.185		2.868	0.646	0.587
Pseudo-first-order irreversible $q_{t}=q_{e}(1-e^{-k_{1}t})$	(26)	104.00	7.221	$k_{1}$ = 2.286 × 10^−1^ (min^−1^)	2.317	0.718	0.696
		154.13	8.963		2.465	0.669	0.643
		194.00	9.860		2.857	0.729	0.708
		217.49	10.359		2.894	0.715	0.693
		281.24	11.524		3.161	0.690	0.666
		309.75	12.185		3.136	0.678	0.653
Pseudo-second-order irreversible $q_{t}=\frac{{q_{e}}^{2}k_{2}t}{1+k_{2}q_{e}t}$	(27)	104.00	7.221	$k_{2}$ = 3.743 × 10^−2^ (g mg^−1^min^−1^)	4.917	0.796	0.780
		154.13	8.963		4.597	0.757	0.738
		194.00	9.860		6.044	0.851	0.840
		217.49	10.359		6.096	0.841	0.828
		281.24	11.524		6.972	0.827	0.814
		309.75	12.185		7.042	0.820	0.807

## Data Availability

The original contributions presented in this study are included in the article. Further inquiries can be directed to the corresponding author.
